# Explainable growth stage classification of cacao (*Theobroma cacao L.*) leaves and key feature visualization using vision transformer and transfer learning

**DOI:** 10.3389/fpls.2026.1885906

**Published:** 2026-09-21

**Authors:** Ezekiel Ahn, Eun-Sung Park, Moon S. Kim, Hangi Kim, Lalit M. Kandpal, Sunchung Park, Seunghyun Lim, Lyndel W. Meinhardt, Byoung-Kwan Cho, Insuck Baek

**Affiliations:** 1 Sustainable Perennial Crops Laboratory, Agricultural Research Service, United States Department of Agriculture, Beltsville, MD, United States; 2 Department of Smart Agricultural Systems, Chungnam National University, Daejeon, Republic of Korea; 3 Environmental Microbial and Food Safety Laboratory, Agricultural Research Service, United States Department of Agriculture, Beltsville, MD, United States; 4 Department of Biosystems Machinery Engineering, Chungnam National University, Daejeon, Republic of Korea

**Keywords:** cacao leaves, deep learning, digital imaging, venation architecture, vision transformer

## Abstract

Accurate and objective plant phenotyping is crucial for optimizing agricultural practices, understanding plant development, and enabling rapid responses to environmental changes. Traditional methods, often relying on visual observation, can be subjective, time-consuming, and may overlook subtle but important differences. This study demonstrates the power of combining digital imaging with deep learning to classify plant material with high accuracy, even when visual differences are minimal. We focused on differentiating between stage D (light green) and stage E (dark green) leaves of cacao (*Theobroma cacao L.*), which are visually very similar in size and overall structure. Using cleared and stained leaves of the SCA 6 genotype to highlight the venation network, we trained a Vision Transformer (ViT) model, a deep learning architecture, on image patches. At the patch level, the model achieved an overall accuracy of 97.03% on an independent test set, with a recall of 96.0% for stage D and 98.3% for stage E. At the whole-leaf level, majority voting correctly classified 14 of 15 independent test leaves (93.3%). Attention maps indicated that image regions containing the midrib and primary lateral veins contributed strongly to classification. These attention maps identify discriminative image regions, but they do not by themselves determine the biological mechanism underlying the signal. The major-vein signal may reflect developmental differences in vein-associated structure, stage-associated differences in Safranin O uptake or optical density, tissue thickness, or a combination of these factors. Because vascular anatomy, lignification, hydraulic conductance, phloem loading, and source–sink status were not directly measured, these mechanisms are treated as hypotheses requiring future anatomical, histochemical, and physiological validation. Thus, this study provides a proof-of-concept for interpretable image-based classification of stage D and stage E leaves within greenhouse-grown SCA 6 cacao. Extension to other cacao genotypes, field-grown plants, independent seasons, staining batches, stress detection, species identification, genotype discrimination, or precision-agriculture deployment will require external validation.

## 1 Introduction

Cacao (*Theobroma cacao L.*), a tropical tree species native to the Americas, has immense global economic and social importance (Díaz-Montenegro, 2019; Hall, 2022). Although cacao originated in the Americas, the majority of the world’s production now comes from tropical regions in West Africa, Southeast Asia, and Latin America. Cacao is the source of cacao beans, the essential ingredient in chocolate, a globally consumed commodity (Wessel and Quist-Wessel, 2015). Beyond the chocolate industry, cacao cultivation provides a livelihood for millions of smallholder farmers, who account for approximately 80% of global production (Wessel and Quist-Wessel, 2015; Bekele and Phillips-Mora, 2019). Sustainable cacao production depends on maintaining high yields and quality, which are directly influenced by crop development and health (Mattalia et al., 2022). Precise growth stage information allows farmers to tailor their management practices, fertilization, irrigation, pest and disease control, and harvesting to the specific requirements of the plant at each stage of development. This targeted approach minimizes resource waste, improves crop productivity, and promotes overall sustainability (Shafi et al., 2019). Traditional methods for assessing cacao growth stage rely heavily on manual observation, a process that is labor-intensive, time-consuming, subjective, and prone to human error. Furthermore, these traditional approaches often fail to capture subtle changes in growth patterns caused by environmental stress or early disease onset, potentially leading to suboptimal management interventions (Haworth et al., 2023).

Advances in image processing and computer vision offer new possibilities for automating and refining plant phenotyping. Early automated approaches utilized techniques like edge detection, segmentation, and color analysis (Weinland et al., 2011; Gevers et al., 2012; Jing et al., 2022). However, these methods often require extensive manual preprocessing and feature engineering, limiting their scalability and robustness in the face of varying field conditions. In recent years, deep learning has revolutionized image analysis in agriculture, offering end-to-end solutions that automatically learn complex features from raw image data. Convolutional Neural Networks (CNNs) have been successfully applied to plant disease classification, growth monitoring, and yield prediction (Toda and Okura, 2019; Teimouri et al., 2018; Nevavuori et al., 2019). More recently, Vision Transformers (ViTs), a technology rooted in Explainable AI (XAI), have emerged as powerful tools for capturing spatial and contextual information in plant images, with reported performance that is competitive with CNN-based approaches in some plant image-analysis tasks (Chitta et al., 2024; Parez et al., 2023).

Despite recent progress in deep learning for plant phenotyping, fine-grained classification tasks involving subtle morphological differences between developmental stages, remains understudied. Traditional visual assessments, often relying on macroscopic features like color, may overlook critical structural variations that provide more precise and objective indicators of plant status. This is particularly relevant in species like cacao (Wickramasuriya and Dunwell, 2018). Cacao trees, which thrive as understory plants in shaded forestry and agroforestry settings, exhibit a unique leaf development characterized by alternate periods of growth and quiescence (flush cycles) (Almeida and Valle, 2007). During a flush, new leaves are produced in rapid succession and expand quickly (Baker and Hardwick, 1973), undergoing a color transition from red or pink to pale green and finally dark green (stages A to E) (Henao Ramírez et al., 2018). While existing literature often categorizes cacao leaf development into these five stages (A-E), with stages D and E distinguished primarily by a shift from light green (D) to dark green (E) (Mejía et al., 2012), this reliance on color may obscure subtle structural differences that are more indicative of physiological maturity. In woody perennials, including cacao, developmental studies have shown that vein density, conduit diameter, and network connectivity can continue to adjust even after the lamina has nearly reached its final size, tightening the hydraulic and mechanical scaffold that supports high photosynthetic capacity and stress tolerance in mature leaves (Almeida and Valle, 2007; Sack and Scoffoni, 2013; Brodribb et al., 2007).

Beyond serving as convenient visual markers, the late D–E stages represent a biologically relevant window in cacao canopy development. Previous physiological and transcriptomic studies have shown that defense-related and primary metabolism genes vary with cacao leaf developmental stage, with mature green leaves deploying a qualitatively different defense machinery compared with younger tissues (Bailey et al., 2005). Recent morphometric studies have also shown that stomatal and chloroplast architectures encode developmental gradients within cacao leaves and that pathogen infection can impose additional stimulus-specific signatures on this shape space (Baek et al., 2025; Lim et al., 2025). These prior observations provide biological context for asking whether cleared and stained leaves contain reproducible image features associated with the D–E transition. However, hydraulic capacity, source–sink status, carbon allocation, immune readiness, and other physiological traits were not directly measured in the present study.

This study addresses this gap by developing a deep learning model by fine-tuning a pre-trained ViT model, leveraging the ViT architecture (Han et al., 2022), to classify cacao leaf growth stages, specifically focusing on the challenging differentiation between stages D and E. We propose a novel approach that combines digital imaging of prepared plant material (cleared and stained leaves) with a ViT model, one of the XAI methods, to achieve highly accurate and interpretable classification. Despite the visual similarity between stages D and E in overall size and color, we hypothesized that consistent structural differences within the leaf architecture would exist and be learnable by a ViT. We further anticipated that by employing attention map visualization, we could gain insights into which specific features the model utilized for classification, potentially revealing previously unappreciated aspects of leaf development.

From a physiological perspective, the late D–E transition may coincide with maturation processes that include changes in vascular organization, mechanical reinforcement, photosynthetic competence, and source–sink status. Previous studies have linked leaf venation traits to hydraulic and photosynthetic function in mature leaves (Sack and Scoffoni, 2013; Brodribb et al., 2007), and the sink-to-source transition is a well-established feature of leaf maturation (Almeida and Valle, 2007; Turgeon, 1989). In the present study, however, these processes were not directly measured. They therefore serve as biological rationale for examining venation-associated image features, rather than as mechanistic conclusions. We hypothesized that cleared and stained leaves would contain reproducible structural or optical features associated with the D–E transition and that these features would be learnable by an interpretable image-classification model.

To investigate these developmental differences, we collected high-quality images of bleached and stained cacao leaves at stages D and E. Image masking was performed using the Segment Anything Model (SAM) (Kirillov et al., 2023), followed by sliding- window technique for data augmentation. A ViT model pretrained on ImageNet-21k was then fine-tuned for classification, and attention map visualization was used to interpret the learned features. Although demonstrated here only for stage D versus stage E classification in greenhouse-grown SCA 6 leaves, this workflow provides a proof-of-concept that could be tested in future studies involving additional cacao genotypes, independent growth environments, field-grown plants, repeated staining batches, and other biological classification tasks.

## 2 Materials and methods

### 2.1 Plant material and growth conditions

To investigate leaf structural differences (e.g. pattern of leaf vein, leaf blade, petiole, epidermis, mesophyll, stomata, etc.) between developmental stages, we used genotype SCA 6, known for its resistance to the oomycete pathogen *Phytophthora megakarya* (Lim et al., 2025). Four clonal trees, derived from semi-hardwood stem cuttings and originally sourced from Pennsylvania State University (2006-2010), were maintained in a USDA-ARS greenhouse in Beltsville, MD, USA. To promote the growth of orthotropic shoots (chupons) for leaf sampling, plants were grown as bentwood plants, a standard propagation technique in cacao. Plants were cultivated under controlled tropical conditions, as described previously (Lim et al., 2025). Briefly, rooted cuttings (after the second leaf flush hardened) were transplanted into a soilless mix of sand:perlite:ProMix BX (2:2:1, v:v:v) and maintained at 29/24 °C (day/night) with 60% relative humidity. A 12-hour photoperiod was provided by natural daylight supplemented with 400W HID lighting. Plants received daily fertigation via an automated irrigation/Dosatron system using Peters’ peat-lite fertilizer (18-8-17 + Mg and micronutrients) and were subsequently re-potted into 2- or 3-gallon pots with the same medium. From September to December 2024, we collected stage D (light green) and stage E (dark green) leaves that were visually selected according to the criteria established by Mejía et al. (2012). After exclusion of structurally defective images and one duplicate image, the final curated dataset contained 95 leaves, consisting of 51 stage D leaves and 44 stage E leaves. Because cacao flushes occur intermittently, target stage D and E leaves were collected opportunistically during repeated greenhouse visits and processed immediately after collection as they became available, either individually or in small batches. The controlled greenhouse environment reduced environmental variability, but the use of a single genotype, four clonal trees, one growth environment, and one sampling period limits the generalizability of the model beyond this experimental setting.

### 2.2 Leaf clearing and staining

Immediately after collection, leaves were prepared for imaging using a modified Safranin O staining protocol adapted from established clearing and staining methods (Johansen, 1940; Vasco et al., 2014). Leaves were first flattened under nylon screening in shallow containers. They were then cleared by immersion in 5% NaOH at 40-54 °C for one to several days, with solution changes as needed, until sufficiently translucent. The leaves were then rinsed thoroughly with water. Bleaching was accomplished with 4.5-5.5% sodium hypochlorite (20s - 10 min), with the process visually monitored to prevent tissue damage, followed by another thorough water rinse. Dehydration was performed using a graded ethanol series (50%, 70%, and 95% for 30 minutes each). Staining was achieved by immersing the leaves in a saturated solution of Safranin O in 95% ethanol for 3 hours. Excess stain was removed by rinsing in 95% ethanol, followed by de-staining in acidified 95% ethanol (3–6 drops of 37% HCl per 100 mL of ethanol; 10–30 min). Finally, the leaves were dehydrated through two changes of 100% ethanol (10 minutes each). All leaves were processed using the same modified clearing and Safranin O staining protocol, reagent concentrations, temperature range, nominal staining duration, dehydration steps, and visual endpoint criteria. The NaOH clearing step was generally performed overnight and extended as needed until the leaf tissue became sufficiently translucent, with solution changes as required. Bleaching and de-staining were endpoint-controlled steps that were visually monitored to obtain comparable vein contrast while minimizing tissue damage and excessive dye removal. Exact durations for these endpoint-controlled steps were not recorded for each individual leaf, and preparation-batch identifiers were not retained as metadata. Therefore, variation in staining intensity, optical density, tissue thickness, or preparation batch cannot be fully excluded as a potential contributor to the image features learned by the model. Future studies should record per-leaf processing times, retain batch identifiers, use batch-balanced experimental designs, and incorporate color/gray calibration or stain-intensity normalization. Representative effects of clearing and Safranin O staining on leaf appearance and venation visibility are shown in **Figure 1**.

**Figure 1:**
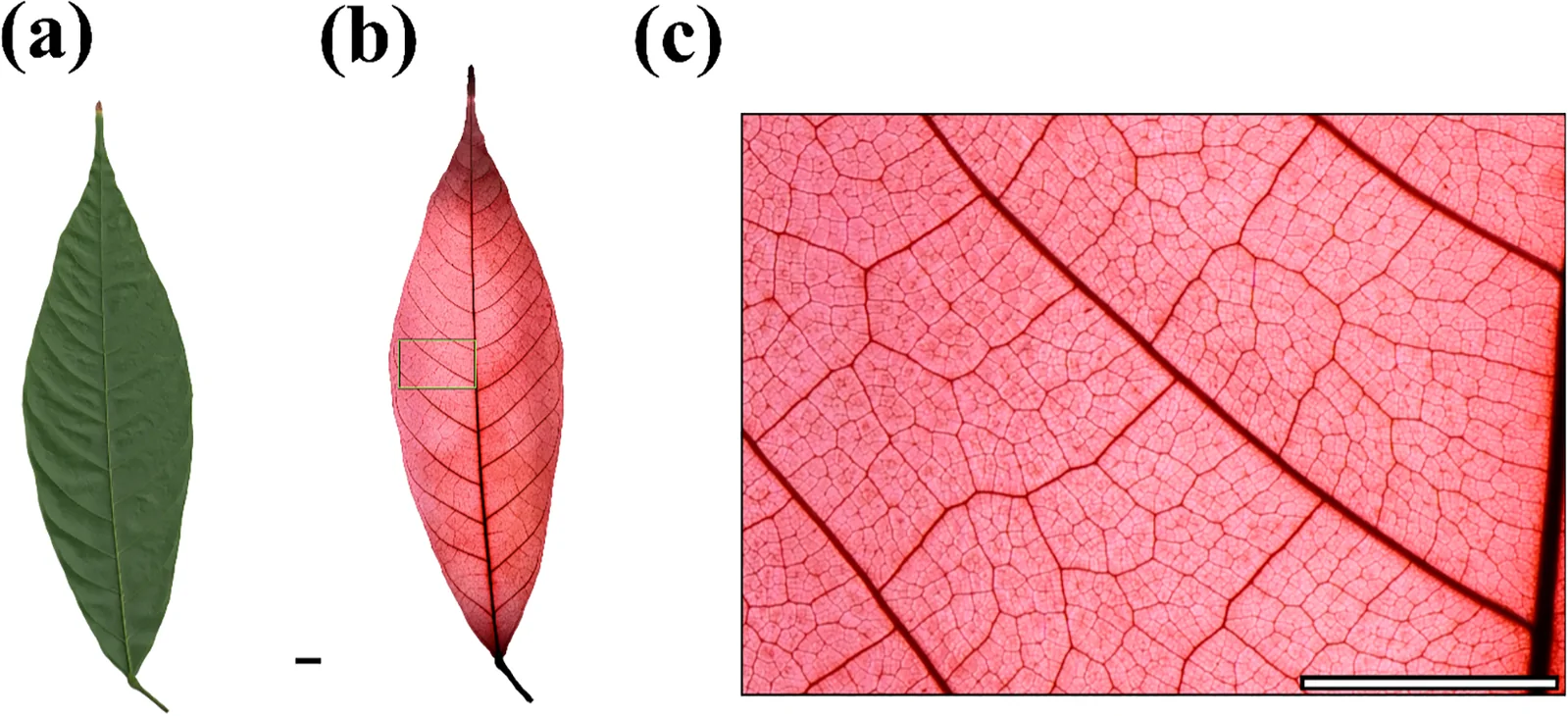
Effects of clearing and staining on the abaxial surface of a stage E cacao leaf. **(a)** A freshly harvested, unprocessed leaf. **(b)** The same leaf specimen after clearing and Safranin O staining, revealing the venation pattern. The green rectangle in **(b)** indicates the region of interest magnified in panel **(c)**. **(c)** High-magnification image of the cleared and stained leaf, showing the intricate details of the major and minor vein network. The black scale bar between panels **(a, b)** is 1 cm. The embedded white scale bar in panel **(c)** also represents 1 cm.

### 2.3 Imaging

Stained leaf images were acquired using a Nikon Z8 camera (Nikon, Tokyo, Japan) equipped with a NIKKOR Z 20 mm f/1.8 S lens and mounted vertically on a Kaiser R2N CP copy stand (Kaiser Fototechnik, North White Plains, NY, USA). The camera head was positioned at the 40-cm mark on the copy-stand scale. For transmitted-light imaging, one Kaiser illumination unit was positioned beneath the specimen, and a transparent glass plate was placed above the light source to support the cleared and stained leaf. The glass surface was positioned approximately 5.5 cm above the stand base. The same camera mount, illumination geometry, glass-plate arrangement, and transmitted-light setup were used throughout image acquisition. Images were recorded in RAW Nikon Electronic Format (NEF). Acquisition parameters were extracted from the original NEF metadata for the source image files used for downstream image processing and model development. Available NEF source files indicated a 20 mm focal length, ISO 100, matrix/pattern metering, no flash, 0 EV exposure compensation, automatic exposure, and automatic white balance. Exposure settings in the available NEF source files ranged from approximately 1/250 to 1/400 s and f/8 to f/11. No independent radiometric calibration using a gray card or color standard was performed, and no manual white-balance calibration was recorded. Therefore, the images were treated as standardized transmitted-light images for structure/venation-based classification rather than as calibrated colorimetric or stain-intensity measurements. A total of 102 stained leaf images were initially acquired. After excluding six structurally defective images and one duplicate stage E image, 95 images were retained for analysis and split at the leaf-image level into training, validation, and test sets before patch generation.

### 2.4 Data preparation

#### 2.4.1 Masking using segment anything model (SAM)

Meta AI’s Segment Anything model (SAM) was used for RGB image masking for this experiment. SAM is a cutting-edge segmentation model designed to automatically generate high-quality masks for a wide range of objects in diverse image datasets (Kirillov et al., 2023). The model is built on Vision Transformer (ViT-h) architecture, which has demonstrated exceptional performance in capturing global dependencies and handling complex visual data (Dosovitskiy et al., 2020). SAM was trained on a massive dataset consisting of 11 million high-resolution images and over 1.1 billion high-quality masks. This extensive training allows SAM to instantly generate high-quality masks for previously unseen objects or image domains (e.g., the specific cacao leaf images used in this study) without any additional fine-tuning. Also, SAM outperforms or even matches existing fully supervised models in various segmentation benchmarks, especially in zero-shot environments (He et al., 2023). This demonstrates that SAM represents state-of-the-art in image segmentation tasks. A key advantage of SAM is its ability to generate multiple mask candidates for a given image, offering flexibility in identifying and selecting the most relevant areas for analysis.

Manual masking inherently suffers from two critical limitations that undermine research reliability: inconsistency and high error rates. First, masking relies heavily on operator subjectivity, such as criteria for handling leaf boundaries and blurred areas. Even within the same operator, masking quality fluctuates depending on fatigue. This severely compromises the consistency of the entire dataset, reducing research reproducibility. In contrast, SAM operates deterministically, generating consistent identical masks for identical inputs and prompts, eliminating these subjective biases. Furthermore, human operators are prone to oversight errors, such as simplifying complex boundaries or missing small but important details, such as subtle background holes between leaves, during tedious, repetitive tasks. SAM operates with pixel-level precision, consistently handling these details, significantly reducing the error rate associated with manual work and enabling the construction of high-quality datasets.

To leverage this capability, rather than selecting the highest-scoring mask candidate, we implemented a method that evaluates all generated mask candidates and selects the most suitable one based on the region of interest (ROI) for the task. In this study, the ROI is defined as the leaf tissue and venation, excluding the black background. This approach ensures more precise and adaptive masking, allowing us to focus on the areas that align with the research objectives. Implementing this SAM-based approach significantly reduced both time and labor compared to manual masking methods. While manual masking often requires substantial time and effort for each image, SAM’s automation drastically reduced the time spent on preprocessing. Additionally, the quality of the masks generated by SAM showed no significant difference from those manually created, demonstrating that SAM is a highly efficient tool for image masking in deep learning applications.

#### 2.4.2 Sliding window data cropping

To prepare the dataset, the high-resolution images were first divided at the image level into training, validation, and test sets (68, 12, and 15 images, respectively). A sliding window technique was then applied separately to each set to generate smaller 448 × 448 pixel patches, so that patches from any single leaf were confined to one set only, preventing information leakage across sets. This technique, a widely used method for data augmentation, involves systematically moving a 448 × 448 pixel window across the entire image (Lashgari et al., 2020). The window was configured with a stride of 112 pixels, resulting in a 75% overlap between consecutive windows. This significant overlap ensures that any given ROI is present in multiple patches, facilitating detailed analysis at a localized level and enhancing the variability of the training data, which is particularly beneficial when the overall dataset size is limited. In cases where the original image dimensions were not exact multiples of 448 pixels, padding was applied to the edges to ensure consistent window sizes without excluding relevant portions of the leaf. After cropping, a masking process was applied to exclude image patches containing only the black background, ensuring that the final dataset consisted solely of patches containing leaf tissue and venation. This preprocessing step was essential for eliminating irrelevant data and enhancing the model’s focus on meaningful features. Through sliding-window augmentation, 39,441 training patches, 6,260 validation patches, and 2,958 test patches were generated. The source leaf-image counts and resulting patch counts for each split are summarized in **Supplementary Table 1**. The sliding-window data cropping procedure is illustrated in **Figure 2**.

**Figure 2:**
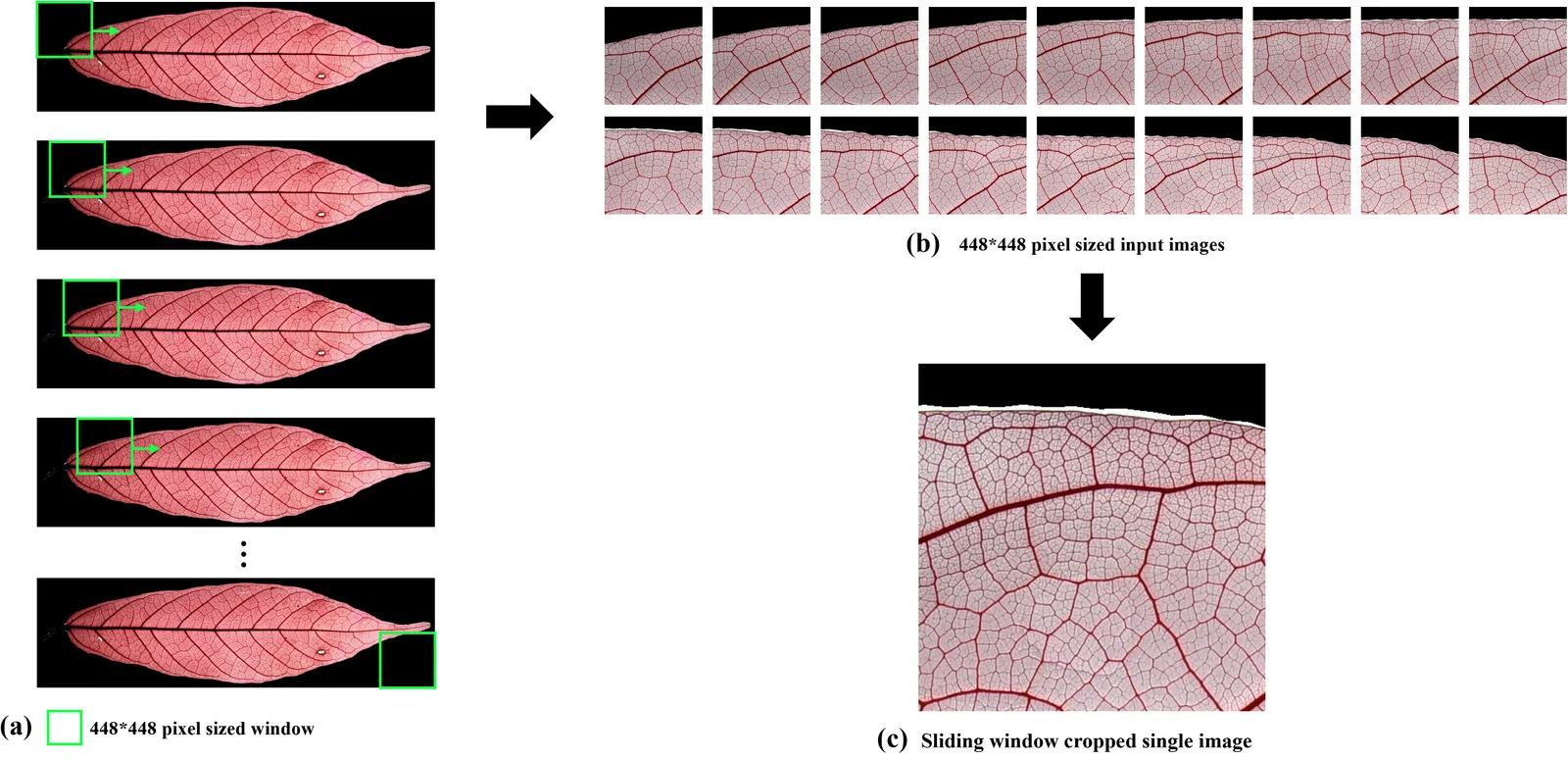
Data augmentation and ROI capture using an overlapping sliding window approach. This figure illustrates the sliding window technique for preparing the cacao leaf image dataset. **(a)** Cleared and stained cacao leaf images with the 448 × 448 pixel sliding window (indicated by green rectangles) overlaid at various positions. The window moves with a stride of 112 pixels, resulting in a 75% overlap between adjacent windows. **(b)** The sliding window generated a series of cropped 448 × 448-pixel image patches, highlighting the overlap. **(c)** A single 448 × 448-pixel image patch, generated through sliding window, containing detailed vein structures, ready for input into the deep learning model. This process ensures that ROIs are captured in multiple patches, enhancing data variability and facilitating localized analysis.

### 2.5 Deep learning

#### 2.5.1 Transfer learning

Using a deep learning model that has already been trained on a sizable, general-purpose dataset for our more specialized objective, we applied transfer learning to improve the model’s performance. Especially when labeled data is scarce, transfer learning enables a model to leverage the insights gleaned from the larger dataset, enhancing generalization and cutting down on training time and computational expense (Ridnik et al., 2021). We chose a ViT that had already been trained on the ImageNet-21k dataset as our foundation model. With over 14 million photos in 21,000 categories, ImageNet-21k is a massive picture classification dataset. The pre-trained ViT has been demonstrated to learn strong and generalizable visual features over a wide range of objects and scenarios, which is why this dataset was selected. ImageNet-21k’s versatility makes it a good choice for offering a solid basis for transfer learning. We used our cacao leaf vein dataset to refine our pre-trained ViT model. By modifying the pre-trained model’s weights with our task-specific data, fine-tuning enables the model to preserve the overall visual information acquired from ImageNet-21k while adapting to the particulars of cacao leaf venation. In the end, this procedure enhanced the model’s performance on our growth-stage classification task.

#### 2.5.2 Vision transformer architecture and training

This study employed the ViT architecture, coupled with transfer learning, for the image-based classification of cacao leaf developmental stages. Unlike traditional CNNs, which rely on local convolutional operations, ViTs adapt the Self-Attention mechanisms of Transformer architecture, initially developed for natural language processing (NLP), to the domain of computer vision. A ViT performs patching and linear projection, it treats an image as a sequence of fixed-size patches, in this study the input image size was 224 × 224 pixels. These patches are linearly embedded into vector representations and processed through successive Transformer layers. This approach enables the model to capture local and global dependencies across the entire image, crucial for understanding the context and relationships between different image regions. A pre-processed cacao leaf image is first divided into a grid of smaller, non-overlapping patches. For the ImageNet-21k model used for this research, each patch is 16 × 16 pixels. This results in a grid of 14 × 14 = 196 patches. Each patch is flattened and linearly projected into a single, long 1D vector. Finally, each of these 196 flattened patch Finally, each of these 196 flattened patch vectors is fed through a standard linear layer, which projects it into the model's high-dimensional embedding space (**Figure 3**).

**Figure 3:**
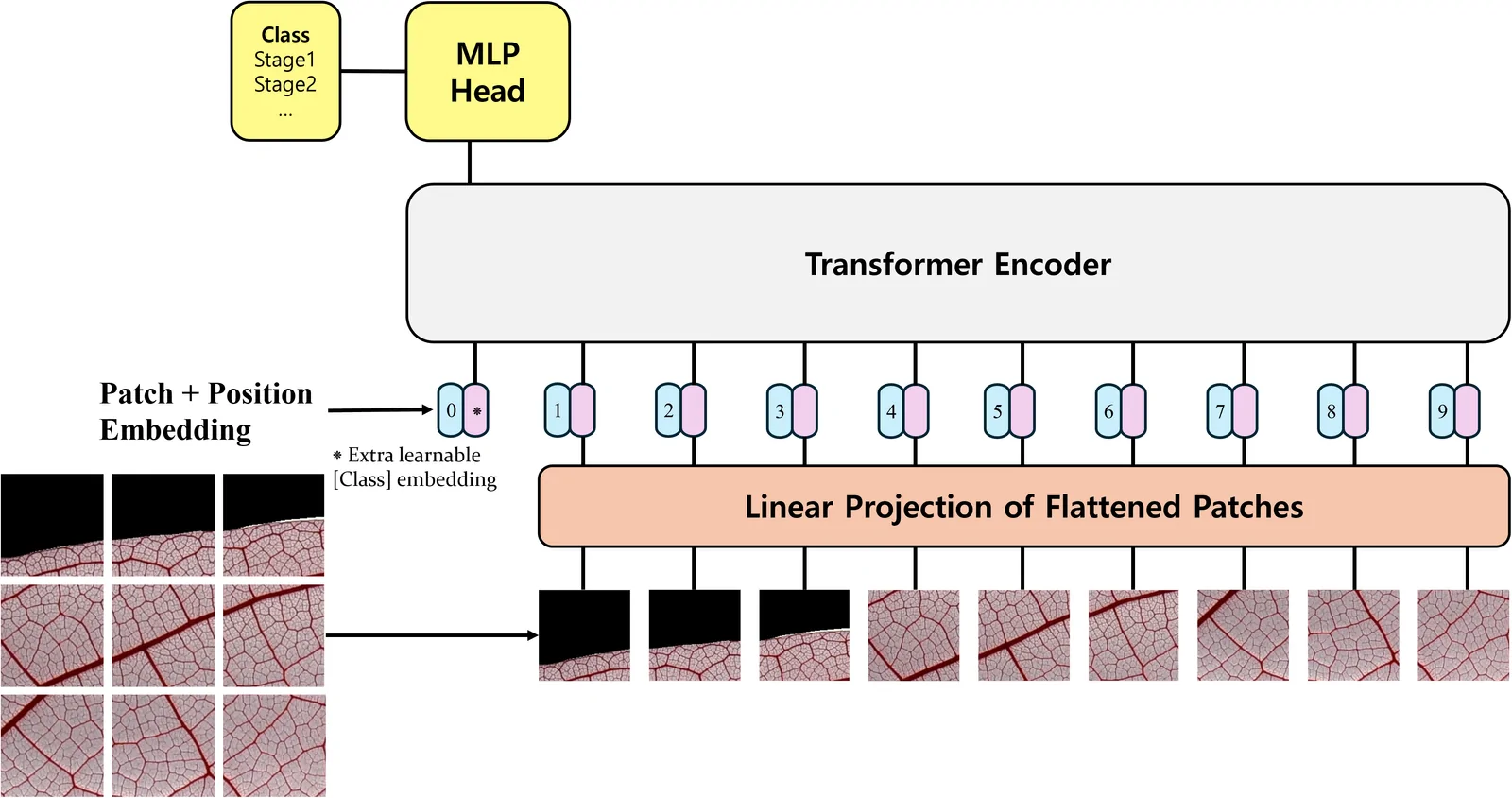
Overview of the ViT model configuration. A pre-processed cacao leaf image is divided into a grid of fixed-size patches. (Middle) Each patch is flattened and linearly projected into a lower-dimensional embedding. Positional embeddings are added to each patch embedding to encode its location within the original image. A learnable class embedding (*) is prepended to the sequence of patch embeddings. This sequence of embeddings is the input to the Transformer encoder. The encoder consists of multiple layers of self-attention and feed-forward networks. The output corresponding to the class embedding is then used by the MLP head to produce the final classification output.

To perform classification, an extra learnable embedding is prepended to the sequence of 196 patches embeddings. This special CLS token is designed to aggregate global information from the entire patch sequence via the self-attention mechanism. Its final output state serves as the sole representation of the image for classification. Additionally, learnable Position Embeddings are appended element-wise to each token in the sequence in order to preserve spatial information that would otherwise be lost during patching. The model can comprehend the 2D structure of the original image thanks to the “Patch + Position Embedding” stage, which gives each patch the required spatial context.

The Transformer Encoder, the model’s central component, is then exposed to the sequence of embedded tokens that results, which includes the CLS token and all patch tokens. A stack of several identical blocks—twelve in the ViT-Base architecture—make up this encoder. Every encoder block has two main sub-layers:

- Multi-Head Self-Attention (MSA): Each token in the sequence can dynamically weigh and integrate data from every other token thanks to this approach. This procedure is specifically used by the CLS token to extract pertinent visual data from every image patch.
- Multi-Layer Perceptron (MLP): Each token representation was subjected to a position-wise, fully connected feed-forward network separately.

To guarantee steady training and efficient information flow across the deep network, Layer Normalization (LN) is used prior to each sub-layer, and residual connections are used following each sub-layer.

Only the output vector that corresponds to the CLS token is extracted after the final encoder block has processed the token sequence. The aggregated representation of the complete image is now included in this single vector, which is input into a final classifier called the “MLP Head.” A straightforward linear layer that projects the vector to the final number of target classes (such as “cacao_D” and “cacao_E”) takes the place of this head for fine-tuning. The final class probabilities are then generated by applying a Softmax function to this output.

The capacity of ViTs to learn long-range relationships without the innate inductive biases of convolutional processes is a significant advantage over CNNs. This makes it possible to extract features in a more flexible and data-driven manner, possibly capturing intricate, domain-specific representations. However, ViTs can be computationally demanding and usually need big datasets for efficient training; we solved these issues with the previously mentioned transfer learning methodology.

Additionally, self-attention techniques are used by Vision Transformers by default, which provides a notable benefit in terms of model interpretability (XAI). The significance of various image regions in the categorization process is measured by these processes. By visualizing these self-attention processes as attention maps, we were able to determine which parts of the leaf images had the greatest bearing on the developmental stage prediction. This representation increases the model’s transparency and fosters confidence in its forecasts.

In conclusion, the ViT architecture was chosen because of its intrinsic transparency transparency and sophisticated feature extraction capabilities (learning long-range dependencies). In order to ensure accuracy and transparency, the model’s decision-making process was made interpretable through the use of attention map visualization.

#### 2.5.3 Attention map visualization

Attention maps, a key feature of ViTs, visually represent the relationships between different image patches. These maps are a direct result of the self-attention mechanism, which is fundamental to how ViTs process images. After an input image is divided into fixed-size patches (treated as tokens), the self-attention mechanism calculates the importance of each patch relative to all others. The attention map, therefore, intuitively reveals which regions of the image the model prioritizes during the decision-making process. A critical advantage of attention maps is their inherent explainability. They directly visualize the parts of the input image that most strongly influenced the model’s predictions, enhancing both the interpretability and reliability of the model’s outputs. This provides valuable insights into the feature-learning process. Compared to visualization techniques used with CNNs, such as Grad-CAM and saliency maps, ViT attention maps offer a more direct and transparent form of interpretability. They are a natural byproduct of the self-attention computation, requiring no additional post-processing. Furthermore, unlike CNNs, which gradually expand their receptive fields, ViTs analyze the global context from the initial layers through direct interactions between all image patches. Because of these strengths, attention maps have widespread use in diverse fields, including crop disease diagnosis (Wang et al., 2021; Alirezazadeh et al., 2023), medical image analysis (Schlemper et al., 2019), and object recognition (Walther et al., 2004). They effectively highlight crucial image regions, significantly improving model interpretability, aiding error diagnosis, and facilitating model design and training improvements. In this study, we utilized attention maps to visually identify cacao leaf features that the ViT model leveraged to predict growth stages.

#### 2.5.4 Model configuration

For this study, we utilized the vit_base_patch16_224_in21k architecture from the timm library, a ViT pre-trained on ImageNet-21k as described in Section 2.5.1. The model’s final classification head was adapted for our binary classification task (stage D vs. stage E) by replacing it with a linear layer containing two output neurons. Training was performed over 70 epochs with a batch size of 32, using the Adam optimizer and a learning rate 1e-4. A Cross-Entropy loss function was employed to optimize model performance. Training was conducted on a single NVIDIA RTX A6000 GPU with 48 GB memory using CUDA version 12.6 on a workstation equipped with an Intel Xeon Gold 6230R CPU and 256 GB DDR4 ECC RAM. Although the computing system included multiple RTX A6000 GPUs, only one GPU was used for model training in this study. Model parameters, optimizer states, the current epoch, and the best validation accuracy were saved after each epoch. This allows training to be resumed from the last saved checkpoint if it is interrupted. Furthermore, the model achieving the highest validation accuracy was saved separately for subsequent inference and deployment. **Table 1** summarizes the training and validation parameters.

**Table 1:** Hyperparameters and configuration for ViT model training and validation.

Parameter	Value
Model architecture	ViT-base (vit_base_patch16_224_in21k)
Pretrained weights	ImageNet-21k
Number of epochs	70
Optimizer	Adam
Learning rate	1e-4
Loss function	Cross-Entropy Loss
Batch size	32
Shuffle	Train: True, Validation: False
Device	Single GPU (NVIDIA RTX A6000, 48 GB memory); CUDA version 12.6

This table summarizes the key hyperparameters and configuration settings used for training and validating the ViT model for cacao leaf growth stage classification. The model was initialized with weights pre-trained on ImageNet-21k and fine-tuned on the prepared dataset of cleared and stained cacao leaf images.

#### 2.5.5 Model performance evaluation

To comprehensively evaluate the performance of our ViT model in classifying cacao growth stages, we employed both Receiver Operating Characteristic (ROC) curve analysis and a confusion matrix analysis. The ROC curve, along with the Area Under the Curve (AUC), provides a visualization and quantification of the model’s ability to discriminate between classes across a range of classification thresholds. This offers a more robust assessment than relying solely on accuracy. The confusion matrix categorizes prediction outcomes into four categories: True Positives (TP), True Negatives (TN), False Positives (FP), and False Negatives (FN). From these values, we derived several key performance metrics: accuracy, precision, recall (sensitivity), specificity, and the F1-score. These metrics are defined as follows:


$$Accuracy=\;\frac{TP+TN}{TP+TN+FP+FN}$$

Precision measures the proportion of true positives among all predicted positives:


$$Precision=\;\frac{TP}{TP+FP}$$

Recall (Sensitivity) reflects the model’s ability to identify actual positives correctly:


$$Recall=\frac{TP}{TP+FN}$$

Specificity represents the model’s effectiveness in identifying actual negatives:


$$Specificity=\frac{TN}{TN+FP}$$

Finally, the F1-score, which combines precision and recall into a single balanced metric, is calculated as follows:


$$F1-score=2\times \frac{Precision\times Recall}{Precision+Recall}$$

In addition, to quantify the uncertainty of the accuracy estimates given the limited test-set size, 95% confidence intervals were computed. At the leaf level, which constitutes the independent sampling unit, the Wilson score interval was used; at the patch level, the interval was estimated for descriptive purposes only, as the overlapping sliding-window patches are not statistically independent. These metrics, taken together, provide a detailed and nuanced understanding of the model’s performance, extending beyond simple accuracy. This comprehensive evaluation confirms the robustness and reliability of the ViT model for classifying cacao leaf growth stages. To assess robustness to the particular data split, leaf-level 5-fold cross-validation was additionally performed: the training/validation pool of 80 leaves was partitioned into five leaf-level folds, while the 15-leaf test set was held out identically across all folds.

## 3 Results

### 3.1 Segment anything

As illustrated in **Figure 4**, the original image must be masked in order to collect images via leaves. Accordingly, only the leaf samples will be used as input data after any irrelevant background features, such as the stand light and components below, have been removed. The performance of the learning model could be impacted severely if these backgrounds are used as input data. Additionally, it may cause confusion for AI learning while making predictions.

**Figure 4:**
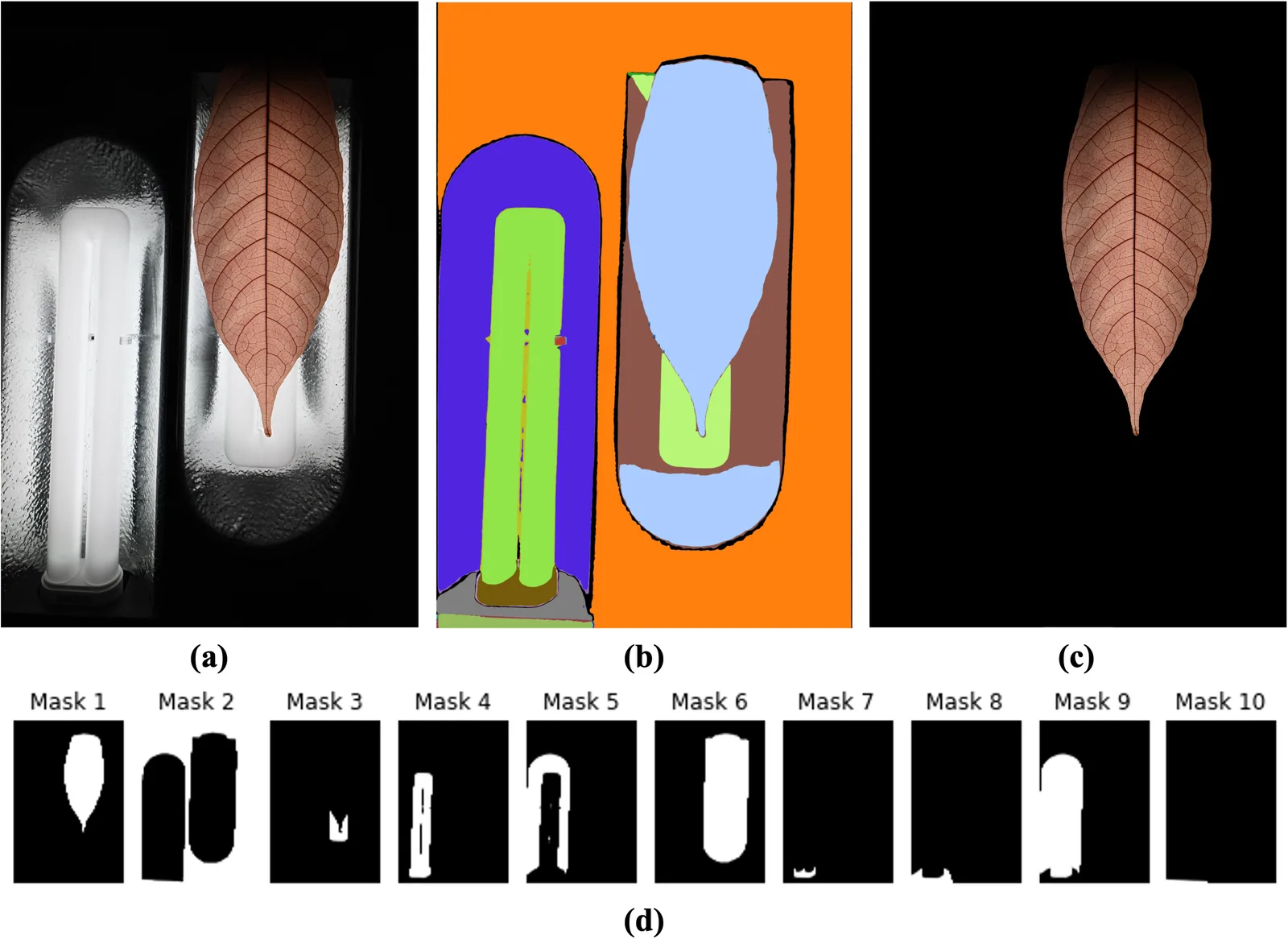
Automated leaf segmentation and masking using SAM. Results of applying SAM for automated ROI masking of a representative cacao leaf image. **(a)** Original RGB image of a cleared and stained cacao leaf. **(b)** Segmentation output from SAM, showing automatically generated mask candidates overlaid on the original image. Different colors represent distinct mask candidates. **(c)** Final masked image, isolating the leaf tissue by applying the selected mask. **(d)** List of top-scored ten mask candidates generated by SAM, ranked by confidence score.

The segmentation masks generated by Meta AI’s SAM for automated region-of-interest (ROI) masking of the cacao leaf images exhibited precision comparable to that of manually created masks (**Figure 4**). To verify this, the SAM masks were quantitatively compared with manually drawn polygon masks on some representative leaf samples using the IoU and Dice coefficient. The SAM masks showed a high level of agreement with the manual masks (mean IoU ≈ 0.96, mean Dice ≈ 0.98). Only the top-ten mask candidates, ranked by their confidence scores, were considered for operational efficiency. These results indicate that SAM provides an accurate and efficient automated segmentation that closely matches manual masking, while removing the labor of manual labeling.

### 3.2 Attention map visualization

Overlaying attention maps, generated by the ViT model using an attention map visualization technique, onto the original input images of cacao leaves revealed the key features driving the model’s classification decisions, which are concentrated on the leaf veins (**Figure 5**). Brighter regions in these attention maps highlight areas of high influence, demonstrating a predominant focus on the venation network of cacao leaves. This localization indicates that major-vein-associated image regions contributed strongly to the model’s classification decisions. However, attention maps should be interpreted as model-attribution outputs rather than direct anatomical or physiological measurements. They identify image regions that influenced classification, but they do not determine whether the discriminative signal arose from vein geometry, staining intensity, optical density, tissue thickness, light scattering, or other correlated features. Thus, the major-vein localization provides a basis for targeted future anatomical and histochemical validation, rather than direct evidence for a specific physiological mechanism. Within these limits, the attention maps support the value of the approach as a proof-of-concept for interpretable image-based phenotyping under controlled experimental conditions.

**Figure 5:**
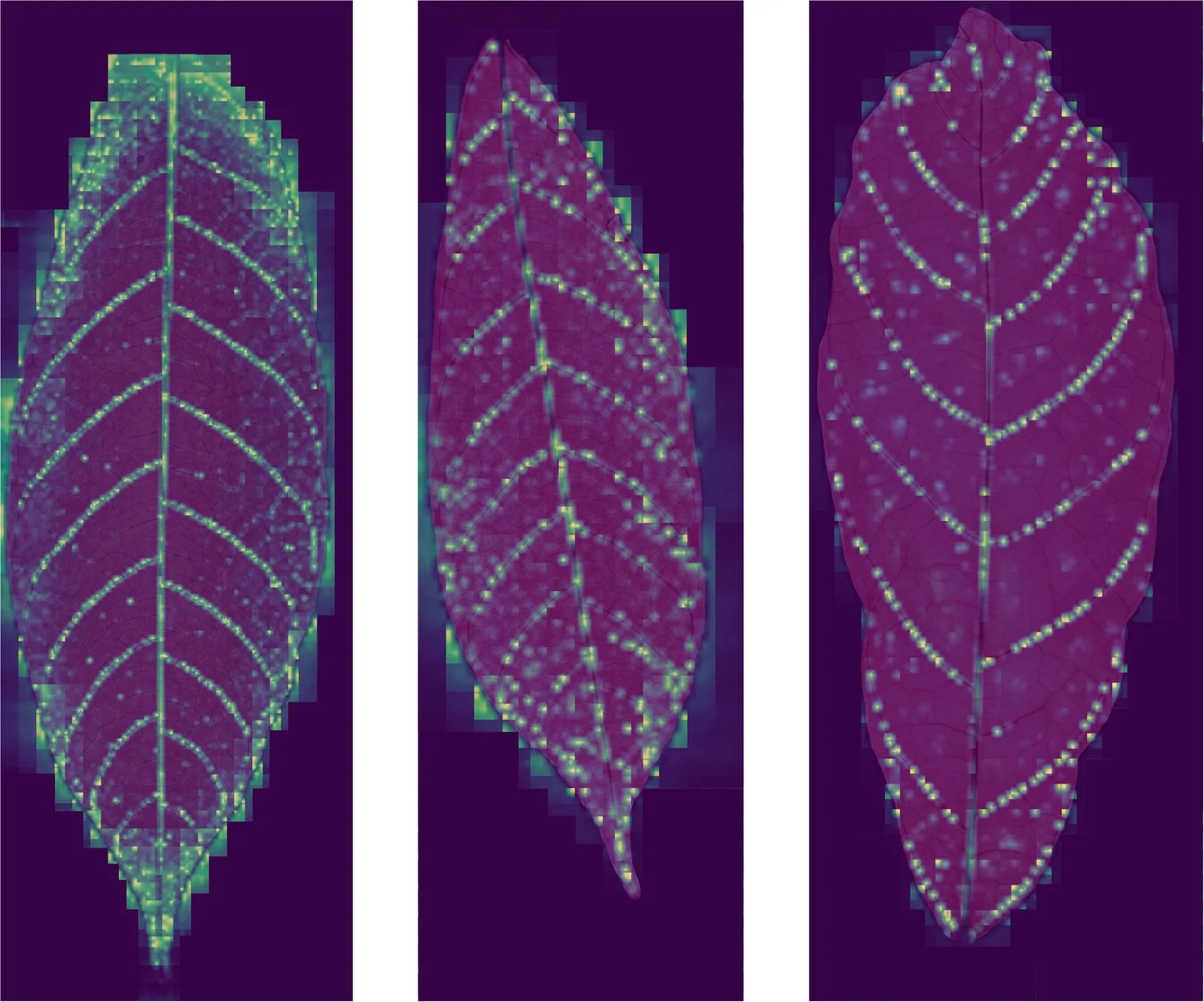
Visualization of attention maps highlighting key features for cacao leaf stage classification. Attention maps overlaid on representative cleared and stained cacao leaf images, illustrating the regions most influential in the ViT model’s classification of developmental stages. (Left) Stage D leaf. (Middle) An example of a Stage E leaf. (Right) Combined presentation of Stage D & E. Highlighted regions indicate areas of higher attention, demonstrating the model’s strong focus on the major veins (midrib and primary lateral veins) for distinguishing between stages D and E.

### 3.3 Leaf-level classification performance and visualization

The ViT model demonstrated strong performance in classifying cacao leaf growth stages (**Figure 6**). In this visualization, correctly classified regions of stage D leaves are shown in red, while correctly classified regions of stage E leaves are shown in green. Misclassified regions are shown in the contrasting color (i.e., stage D regions classified as stage E are green, and stage E regions classified as stage D are red). For the background image that does not include the leaf components, it was arbitrarily visualized as class E in the figure below, but it did not influence the prediction.

**Figure 6:**
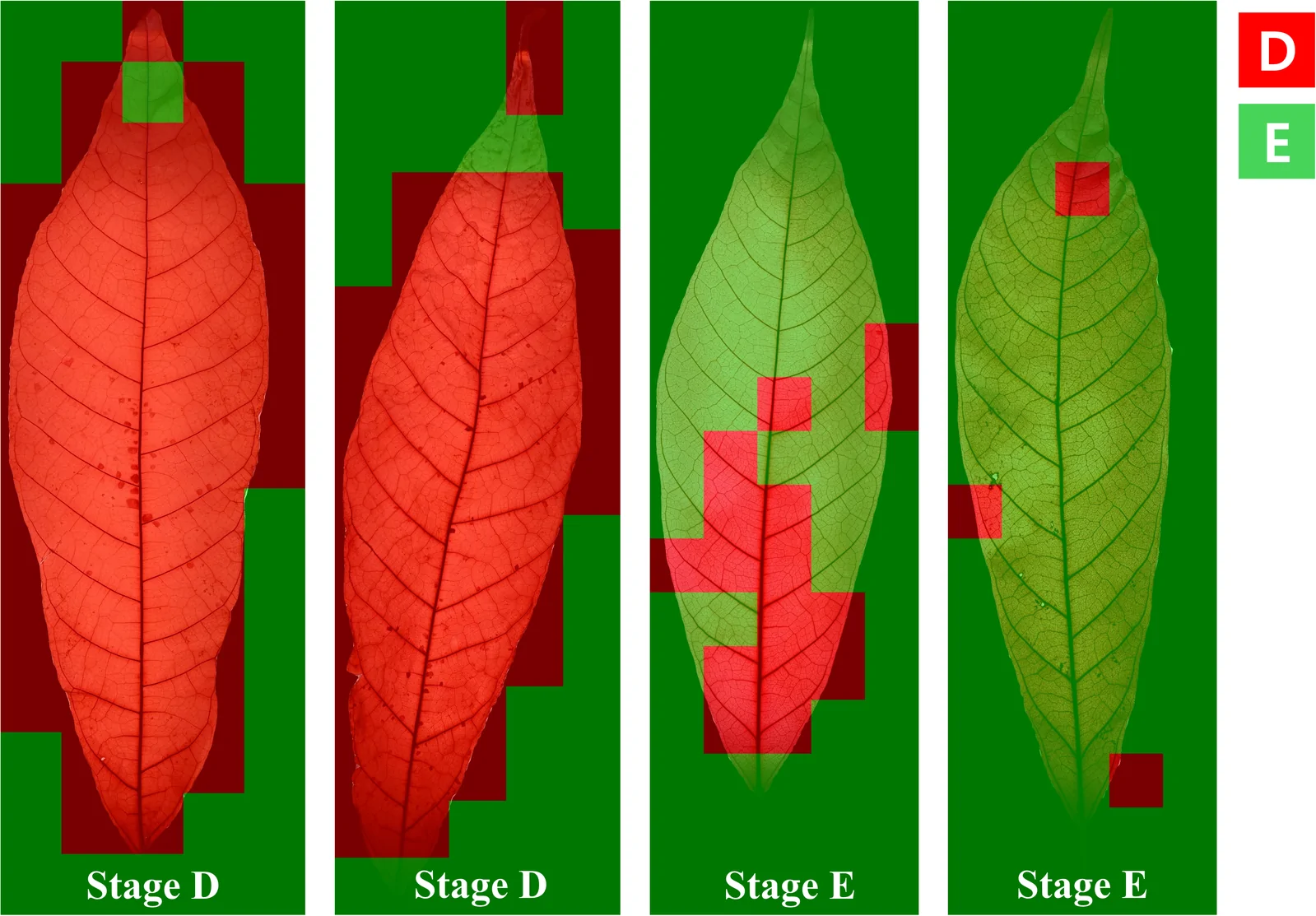
Patch-level classification results for cacao leaf developmental stages. Visualization of the ViT model’s patch-level classification results on representative cacao leaf images. (Left two panels) Stage D leaves. (Right two panels) Stage E leaves. Red regions indicate areas correctly classified as stage D. Green regions indicate areas correctly classified as stage E. Misclassified regions are shown in the contrasting color. The predominantly red coloration of the stage D leaves and green coloration of the stage E leaves visually demonstrates the high classification accuracy achieved by the model. The final image-level classification was determined by a majority vote across all patches within each image.

As shown in **Figure 6**, the majority of leaf regions were correctly classified. The final prediction for each test image was determined using a majority voting approach: predictions from all individual patches within an image were aggregated, and the class with the most patch-level predictions was assigned as the overall image classification. For this whole-leaf evaluation, each test leaf was partitioned into non-overlapping patches before majority voting, whereas the patch-level metrics reported in Section 3.4 were calculated from the overlapping sliding-window patches. It is important to note that the background regions, which do not contain leaf tissue, were included in the visualization for completeness but were excluded from the classification process itself. This approach resulted in accurate classification of 14 out of 15 test images, yielding an overall accuracy of approximately 93.3%. Using the Wilson score interval, this leaf-level accuracy corresponds to a 95% confidence interval of 70.2–98.8%; the wide interval reflects the uncertainty associated with the small number of independent test samples (n = 15).

Close examination of the single misclassified sample in the test set (**Figure 7**) revealed potential contributing factors. This particular leaf exhibited significant physical damage, including tearing and structural irregularities. This damage likely introduced ambiguity into the feature extraction process, leading to an even prediction split (50:50) between classes D and E. This suggests that, in the absence of such damage, the model would likely have achieved a more definitive classification. This case underscores the importance of sample integrity for optimal classification accuracy and highlights the need for future research. Specifically, developing classification strategies that are robust to damaged or atypical samples is a valuable direction for further investigation. Overall, the ViT model’s demonstrated high accuracy and consistent performance in growth-stage classification. Combined with the interpretability provided by attention-map visualization, these results support the feasibility of whole-leaf D–E classification in the present controlled SCA 6 greenhouse dataset.

**Figure 7:**
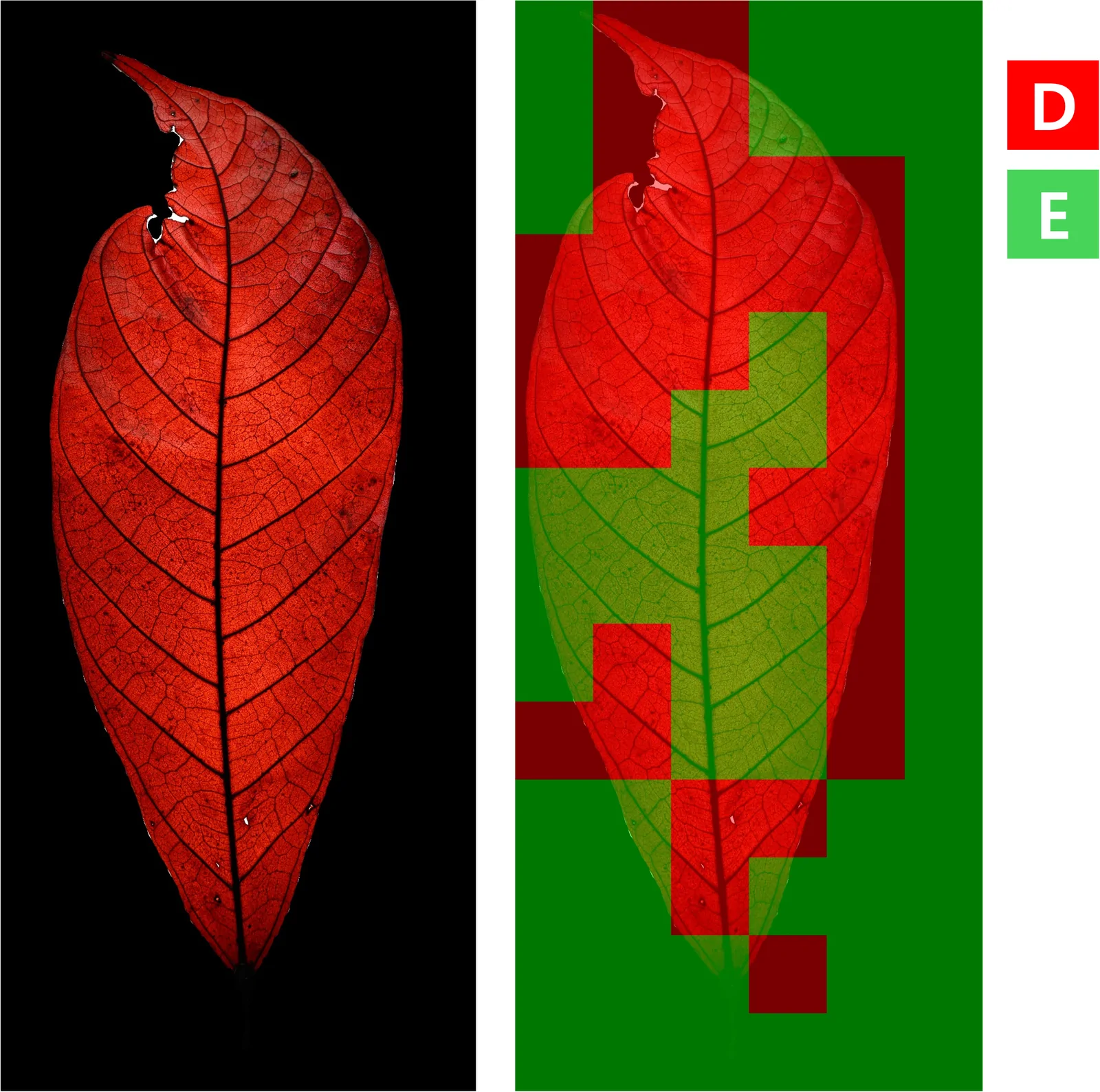
Misclassified cacao leaf sample exhibiting physical damage. Visualization of the single misclassified cacao leaf sample. (Left) The original cleared and stained leaf image, showing significant tearing and structural damage. (Right) The patch-level classification results overlaid on the leaf image. Red regions indicate areas classified as stage D, while green regions indicate areas classified as stage E. The mixed classification (approximately 50% D and 50% E) across the leaf area, unlike the consistent classifications in **Figure 6**, is likely due to the disruption of the venation pattern caused by the physical damage. This highlights the impact of sample quality on classification accuracy.

For the CNN backbone evaluation, EfficientNet-B0 and ResNet-50 were each trained and evaluated once using the 15 test leaves. Following majority voting of their patch-level predictions, both CNN models correctly classified 14 of the 15 leaves, corresponding to a leaf-level accuracy of 93.3%. No leaf-level misclassification was observed for either architecture. Thus, although patch-level errors occurred, they did not alter the final whole-leaf classifications. These CNN results provide a descriptive comparison based on a single training run for each architecture and a limited number of independent test leaves.

Overall, the ViT, EfficientNet-B0, and ResNet-50 models demonstrated strong whole-leaf classification performance within the present controlled SCA 6 greenhouse dataset. The patch-level visualization also provided information about the spatial distribution of correct and incorrect predictions within each leaf. Nevertheless, the small number of test leaves and the use of a single training run for each model limit the extent to which these findings can be generalized.

### 3.4 Patch-level classification performance and evaluation metrics

The confusion matrix in **Figure 8** summarizes the classification performance of the ViT model on the test dataset. The model achieved an overall accuracy of 97.03%, indicating strong predictive capability. Of the total 2,958 test image patches, 1,564 (52.8%) were correctly classified as stage D, and 1,306 (44.2%) were correctly classified as stage E. Misclassifications were relatively low: 66 (2.2%) stage D patches were incorrectly classified as stage E, and 22 (0.7%) stage E patches were incorrectly classified as stage D. These results demonstrate the model’s ability to effectively differentiate between the two cacao growth stages, with a high degree of precision and consistency. The corresponding Wilson 95% confidence interval for the patch-level accuracy (97.03%) was 96.4–97.6%; however, because the patches were generated using overlapping sliding windows and are therefore not independent, this interval is affected by pseudo-replication and should be interpreted as a descriptive estimate rather than as an independent-sample estimate of generalization.

**Figure 8:**
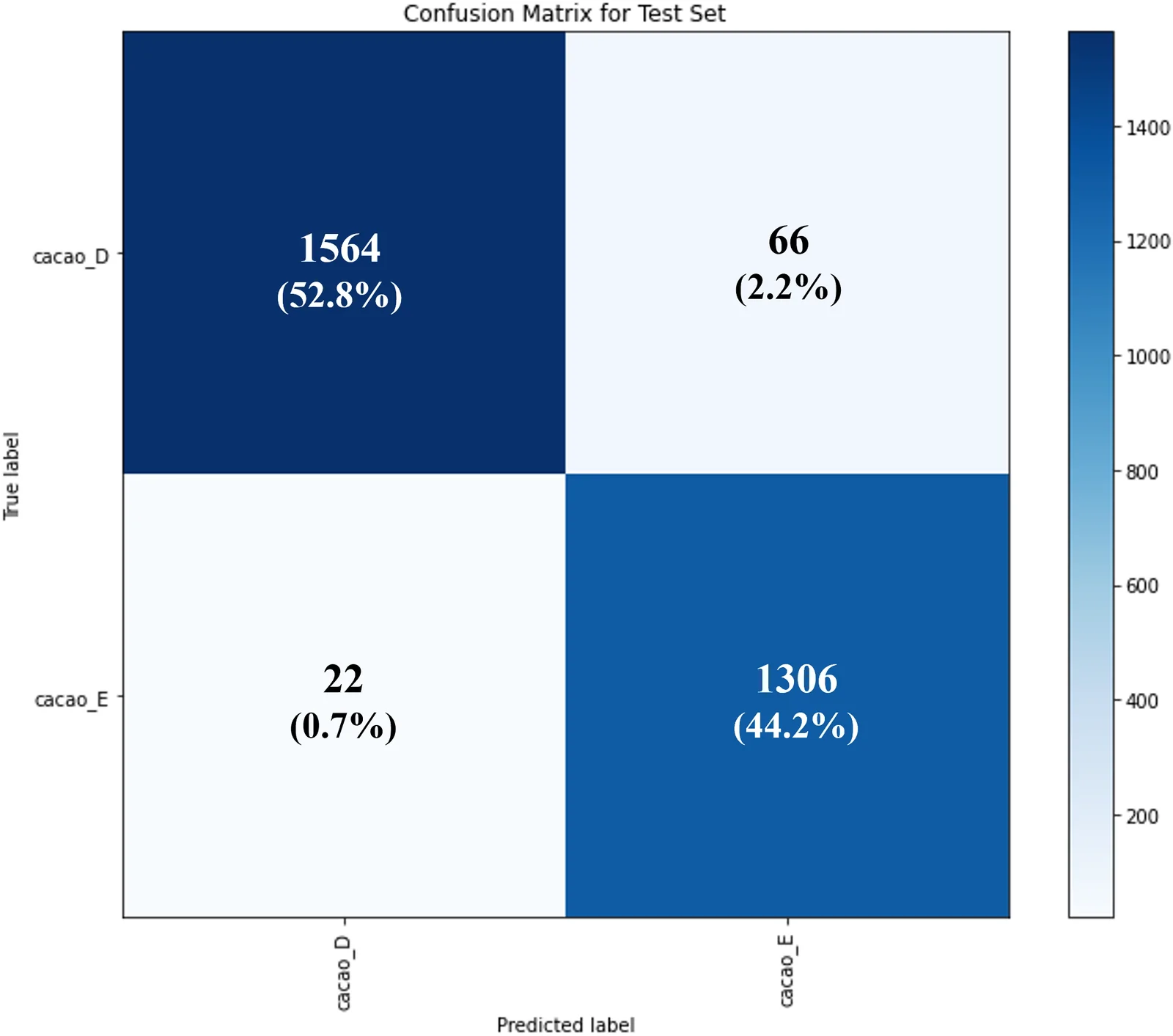
Confusion matrix of cacao leaf growth stage classification result (Patch-Level). Confusion matrix summarizing the patch-level classification performance of the ViT model on the test dataset. The rows represent the true (actual) growth stages (D and E), and the columns represent the predicted growth stages. Each cell contains the number and percentage of image patches falling into that true/predicted category. The diagonal elements (1564 and 1306) represent correct classifications (True Positives and True Negatives), while the off-diagonal elements (66 and 22) represent misclassifications (False Positives and False Negatives). The overall accuracy, as derived from this matrix, is 97.03%. The color gradient visually represents the magnitude of the values, with darker shades of blue indicating higher counts.

The model demonstrated strong overall classification performance, achieving an accuracy of 97.03% (**Table 2**). For stage D, the model achieved a precision of 98.61%, indicating that a high proportion of patches classified as stage D were correct. The recall (sensitivity) for stage D was 95.95%, demonstrating the model’s ability to accurately identify the actual stage D patches. The specificity for stage D was 98.34%, showing the model’s effectiveness in correctly identifying patches *not* belonging to stage D. The F1-score, a balanced measure of precision and recall, was 97.26% for stage D. For stage E, the precision was 95.19%, the recall was 98.34%, and the specificity was 95.95%. The F1-score for stage E was 96.74%. These metrics, taken together, demonstrate the ViT model’s strong and reliable predictive capability for classifying cacao leaf growth stages. These metrics indicate strong patch-level classification performance within the held-out test set. However, because overlapping sliding-window patches are not statistically independent, patch-level metrics are interpreted as descriptive support for the leaf-level majority-vote classifier rather than as independent biological replicates. The leaf-level result remains the more conservative measure of whole-leaf classification performance.

**Table 2:** Performance metrics for cacao leaf growth stage classification.

Metric	Stage D	Stage E
Accuracy	0.970	
Precision	0.986	0.952
Recall	0.960	0.983
F1 Score	0.972	0.967
Specificity	0.983	0.960

Summary of performance metrics for the ViT model’s classification of cacao leaf growth stages D and E, calculated at the patch level. Metrics are derived from the confusion matrix (**Figure 8**). Accuracy represents the overall proportion of correctly classified patches. Precision, recall, F1-score, and specificity are presented separately for each stage.

The CNN backbone evaluation was conducted using test patches derived from 15 leaves. EfficientNet-B0 achieved a patch-level accuracy of 89.25% and a macro-F1 score of 0.8892. Its recall values were 97.4% for stage D and 79.4% for stage E. ResNet-50 achieved a patch-level accuracy of 91.99% and a macro-F1 score of 0.9186, with recall values of 95.8% for stage D and 87.4% for stage E. EfficientNet-B0 therefore showed slightly higher recall for stage D, whereas ResNet-50 showed more balanced recall across the two growth stages.

In this evaluation, the patch-level accuracy of ResNet-50 was 2.74 percentage points higher than that of EfficientNet-B0. This difference represents a descriptive comparison of the observed results and should not be interpreted as evidence of a statistically established performance difference or as an estimate of variation across repeated training runs.

### 3.5 Evaluation of classification performance using ROC and precision-recall curves

To further assess the performance of the ViT model in differentiating between cacao leaf growth stages D and E, we conducted Receiver Operating Characteristic (ROC) and Precision-Recall (PR) curve analyses (**Figure 9**). The ROC curve (**Figure 9a**) yielded an Area Under the Curve (AUC) value of 0.98, demonstrating excellent discriminatory ability. This high AUC indicates that the model consistently achieves high sensitivity (true positive rate) while maintaining a low false positive rate across a range of classification thresholds. The PR curve (**Figure 9b**) provided additional insights, particularly relevant in scenarios with potential class imbalances. The Average Precision (AP) derived from the PR curve was 0.97, reflecting a strong balance between precision (the proportion of correctly predicted positives among all predicted positives) and recall (the proportion of actual positives correctly identified). This high AP value indicates that the model consistently achieves high precision and high recall across different probability thresholds. Collectively, the high AUC and AP values support the model’s ability to distinguish stage D and stage E patches within the held-out test set, while broader generalization will require validation with additional independent leaf-level datasets.

**Figure 9:**
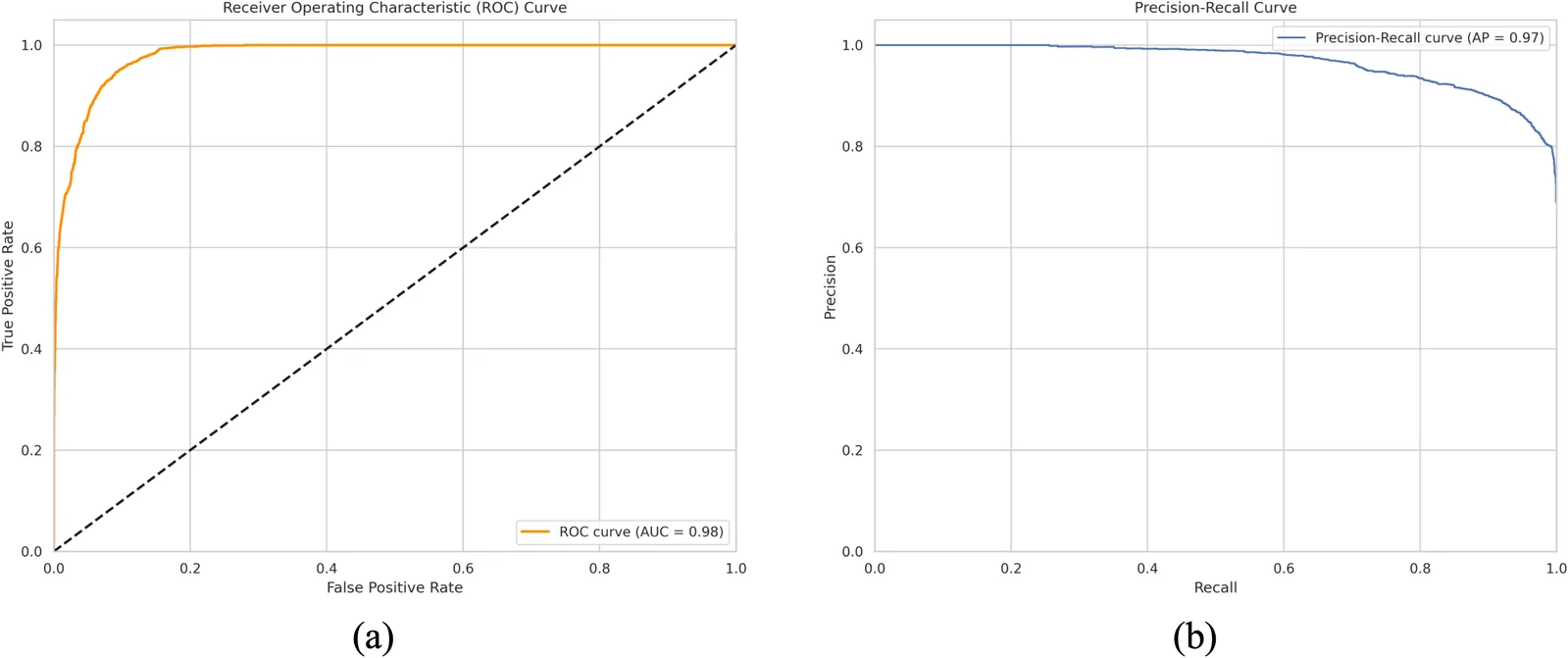
ROC and precision-recall curves for cacao leaf growth stage classification. Evaluation of the ViT model’s performance in classifying cacao leaf growth stages D and E using ROC and PR curves. **(a)** ROC curve, plotting the True Positive Rate (Sensitivity) against the False Positive Rate at various classification thresholds. The AUC is 0.98, indicating excellent discriminatory ability. The dashed diagonal line represents random chance (AUC = 0.5). **(b)** PR curve, plotting Precision against Recall at various classification thresholds. The AP is 0.97, indicating a strong balance between precision and recall across different thresholds. Both the high AUC and AP values demonstrate the model’s robust performance in distinguishing between the two leaf growth stages.

### 3.6 Cross validation

Leaf-level five-fold cross-validation yielded consistent performance across the five folds and across the three architectures. The validation leaf-level accuracy was 93.8 ± 4.2% for all three models, while the patch-level accuracy was 91.2 ± 5.6% for EfficientNet-B0, 91.5 ± 5.3% for ResNet-50, and 91.9 ± 5.0% for ViT-B/16. The identical leaf-level values across the three models reflect that all architectures misclassified the same small subset of leaves within the same-batch validation pool, whereas their patch-level accuracies differed slightly. The small variation across folds and the similar performance of the three architectures indicate that the classification results are not strongly dependent on a single particular data split. The final metrics reported in Sections 3.3 -3.4 were obtained using the independent 15-leaf test set, which remained completely excluded from all cross-validation folds.

## 4 Discussion

This study investigated the potential of deep learning to differentiate between late-stage (D and E) cacao leaves, a task traditionally reliant on subjective visual assessments of leaf characteristics. The findings demonstrated that a ViT model, trained on images of cleared and stained cacao leaves, accurately classified these stages, achieving an overall accuracy of 97.03% on the test dataset. This high level of accuracy was attained despite the subtle visual differences between stages D and E, suggesting that underlying structural features—particularly within the leaf venation network—were successfully captured and learned by the model. Although the confusion matrix revealed minor misclassification occurrences, with precision and recall balanced closely between both classes (Stage D: precision 98.61%, recall 95.95%; Stage E: precision 95.19%, recall 98.38%), the overall strong performance of the model confirms its robust capability in effectively classifying cacao leaf growth stages. These results indicate that cleared and Safranin O-stained images contain reproducible image features sufficient for distinguishing stage D and stage E leaves within the present controlled SCA 6 dataset. Prior work indicates that cacao flush dynamics and leaf venation are closely related to leaf development and function (Almeida and Valle, 2007; Sack and Scoffoni, 2013). However, in the present experiment, these references serve as biological context rather than direct mechanistic evidence. The experiment does not distinguish whether the model’s discriminative features reflect true developmental re-patterning of venation geometry, stage-associated differences in stain uptake or optical density, tissue thickness, light scattering, or a combination of these factors. Therefore, we interpret the major-vein signal as an image-level marker associated with the D–E transition, not as direct evidence of vascular maturation, hydraulic function, or source–sink physiology.

Several image-based plant classification studies provide useful contextual benchmarks for the present results. For example, prior studies have reported high classification performance for plant disease recognition, nutrient-deficiency classification, grapevine species classification, and plant leaf family classification using CNNs, hybrid CNNs, MobileNet/VGG-based models, artificial neural networks, or support vector machines (Chen et al., 2020; Sherline Jesie and Godwin Premi, 2024; Elkassar, 2024; Sharma et al., 2024). These studies differ from the present work in species, task, image type, dataset size, and number of classes, and therefore do not constitute a controlled comparison of model architectures. Rather, they provide context showing that image-based learning can achieve high performance across plant phenotyping tasks. In the present study, the key point is that cleared-leaf imaging combined with an interpretable ViT model enabled classification of visually similar cacao leaf stages, while attention-map visualization indicated that major-vein-associated regions contributed strongly to the prediction.

The attention maps generated by the ViT indicated that classification decisions were strongly associated with the midrib and primary lateral veins, with comparatively less emphasis on inter-veinal regions or minor venules. This localization is consistent with the established importance of leaf venation architecture for mechanical support, transport efficiency, photosynthetic function, and drought-related performance (Roth-Nebelsick et al., 2001; Sack and Scoffoni, 2013; Brodribb et al., 2007). It is also consistent with previous deep learning work showing that venation features can be important for plant classification (Lee et al., 2017). However, attention maps are not anatomical measurements, and Safranin O staining can accentuate cell-wall-rich and thicker tissues such as the midrib and primary veins. Therefore, we cannot exclude the possibility that the model used differences in stain accumulation, optical density, tissue thickness, or light scattering, rather than—or in addition to—true venation-geometry differences. We therefore interpret the major-vein attention signal cautiously as a candidate image marker that requires validation by quantitative vein morphometry, microscopy, and histochemical analysis.

Several physiological mechanisms could plausibly contribute to stage-associated differences in major-vein image features, but these remain hypotheses. Candidate processes include the sink-to-source transition of maturing leaves (Turgeon, 1989), increased cell-wall deposition or lignification (Bonawitz and Chapple, 2010; Donaldson, 2001), changes in xylem or phloem structure associated with mature leaf function (Brodribb et al., 2007; Bertamini and Nedunchezhian, 2003), altered tissue thickness, or shifts in residual phenolic or other secondary compounds that influence Safranin O staining and light scattering (Cheynier et al., 2013). Because these traits were not measured directly in the present study, future work should combine cleared-leaf imaging with microscopy, quantitative vein morphometry, lignin/cell-wall staining, and physiological measurements to determine whether the image features used by the model correspond to specific developmental mechanisms.

This research represents a significant advancement, demonstrating the potential of deep learning to move beyond traditional visual assessments in plant phenotyping. The ability of our model to discern subtle differences between developmental stages based primarily on major vein features is notable, particularly considering that leaf venation networks, in general, exhibit a complex mixture of scaling behaviors, with some aspects following characteristic scales and others exhibiting scale-free properties (Price et al., 2012). More broadly, this study demonstrates a proof-of-concept workflow for combining cleared-leaf imaging, ViT-based classification, and attention-map visualization in a controlled phenotyping setting. Leaf venation networks exhibit complex scaling and structural properties (Price et al., 2012), and related image-based approaches have been explored for plant disease detection, drought-stress classification, nutrient-deficiency assessment, genotype-to-phenotype prediction, species identification, and developmental monitoring (Parez et al., 2023; An et al., 2019; Azimi et al., 2021; Danilevicz et al., 2022; Chouhan et al., 2024; Gao et al., 2020; Tong et al., 2022; Samiei et al., 2020). These studies support the broader motivation for image-based phenotyping, but they do not validate general deployment of the present model. Whether the same workflow can support genotype discrimination, species identification, stress detection, or classification of other developmental stages remains to be tested directly. Such applications will require independent datasets that include multiple genotypes, species, environmental conditions, staining batches, imaging sessions, and field-grown samples. For cacao agronomy and breeding, an objective image-based marker of the D–E transition could become useful only after external validation. In future studies, similar classifiers should be tested across independent greenhouse and field-grown material, additional genotypes, repeated seasons, and independent staining/imaging batches before they are considered for field- or drone-based phenotyping platforms.

A further limitation is that the clearing/staining and imaging workflow was standardized but not fully radiometrically controlled. Leaves were collected over repeated greenhouse visits as stage D and stage E leaves became available during flush cycles and were processed immediately using the same protocol. However, exact per-leaf processing times, preparation-batch identifiers, fixed manual exposure settings, and color/gray calibration targets were not recorded. Therefore, residual effects of staining intensity, optical density, tissue thickness, preparation batch, automatic exposure, or automatic white balance cannot be fully excluded. Future validation should include batch-balanced staining designs, per-leaf processing logs, fixed camera exposure and white-balance settings, radiometric calibration, and independent anatomical measurements to determine whether the model relies primarily on venation geometry, stain uptake, optical density, or a combination of these features.

Looking forward, this proof-of-concept work motivates several future research directions. Larger and more diverse datasets encompassing different cacao genotypes, independent seasons, field-grown plants, and varying environmental conditions will be required to test the generalizability and robustness of the workflow. Further investigation using microscopy, quantitative vein morphometry, histochemical staining, and physiological assays could help determine whether the image features highlighted by the model correspond to specific structural or chemical differences at the cellular level. Controlled comparisons between ViTs, CNN baselines, classical feature-based classifiers, and human visual classification would also clarify whether the ViT architecture provides a performance advantage beyond the cleared-leaf imaging pipeline itself. Cacao-focused machine-learning studies in fermentation, post-harvest management, canopy estimation, yield prediction, aroma analysis, and floral traits illustrate the broader interest in computational approaches for cacao research (Tan et al., 2019; Ferran et al., 2024; Duarte-Carvajalino et al., 2021; Gamboa et al., 2019; Fuentes et al., 2019; Lim et al., 2025). These studies provide context for future applications, but external validation remains necessary before the present workflow can be generalized beyond the controlled SCA 6 dataset.

## 5 Conclusion

This study provides a proof-of-concept demonstration that cleared and Safranin O-stained cacao leaves contain reproducible image features that can be used by a ViT model to distinguish stage D and stage E leaves within a controlled SCA 6 greenhouse dataset. At the whole-leaf level, the model correctly classified 14 of 15 independent test leaves, while patch-level analysis showed high classification performance. Attention-map visualization indicated that the model placed strong emphasis on the midrib and primary lateral veins, suggesting that major-vein-associated image features contributed substantially to the classification decision. The biological basis of these image features remains unresolved. Because vascular anatomy, lignification, hydraulic conductance, phloem loading, and source–sink status were not directly measured, the observed major-vein signal should be interpreted as a hypothesis-generating image marker rather than direct evidence of a specific developmental mechanism. In addition, variation in clearing, Safranin O staining, tissue thickness, optical density, preparation batch, automatic exposure, and automatic white balance may have contributed to the features learned by the model. These factors should be controlled more rigorously in future studies through per-leaf processing logs, staining-batch controls, fixed imaging settings, radiometric calibration, stain-intensity normalization, and independent anatomical validation. The generalizability of the model is limited by the present dataset, which included one genotype, four clonal greenhouse-grown trees, one sampling period, and one preparation/imaging workflow. Therefore, applications to other cacao genotypes, field-grown plants, independent seasons, different staining batches, stress detection, genotype discrimination, species identification, or precision-agriculture deployment require external validation. Within these limits, the study establishes a useful starting point for interpretable, image-based classification of subtle cacao leaf developmental stages and provides a framework for future validation across broader biological and agricultural contexts.

## Data Availability

The raw data supporting the conclusions of this article will be made available by the authors, without undue reservation.

## Supplementary material

Supplementary Table 1 Leaf-level data split and resulting patch numbers. Source leaf images were split before patch generation, ensuring that all patches from a given leaf were confined to a single dataset split.
