# Supplementary material for: Explainable growth stage classification of cacao (Theobroma cacao L.) leaves and key feature visualization using vision transformer and transfer learning
Source: Front Plant Sci. 2026 Sep 21;17:1885906. doi: 10.3389/fpls.2026.1885906 (PMC13635337; doi:10.3389/fpls.2026.1885906)
Supplement: Supplementary file 1 [file Table1.docx]

**Supplementary Material**

Explainable growth stage classification of cacao (Theobroma cacao L.) leaves and key feature visualization using vision transformer and transfer learning

# Supplementary Table 1

Leaf-level data split and resulting patch numbers. Source leaf images were split before patch generation, ensuring that all patches from a given leaf were confined to a single dataset split.

| **Dataset split** | **Source leaf images (n)** | **Image patches (n)** |
| --- | --- | --- |
| Training | 68 | 39,441 |
| Validation | 12 | 6,260 |
| Test | 15 | 2,958 |
| Total | 95 | 48,659 |

The counts correspond to the data-preparation description in the author proof: 68 training leaves generated 39,441 patches, 12 validation leaves generated 6,260 patches, and 15 test leaves generated 2,958 patches.
